# A comparison between two semantic deep learning frameworks for the autosomal dominant polycystic kidney disease segmentation based on magnetic resonance images

**DOI:** 10.1186/s12911-019-0988-4

**Published:** 2019-12-12

**Authors:** Vitoantonio Bevilacqua, Antonio Brunetti, Giacomo Donato Cascarano, Andrea Guerriero, Francesco Pesce, Marco Moschetta, Loreto Gesualdo

**Affiliations:** 1 Department of Electrical and Information Engineering (DEI), Polytechnic University of Bari, Italy, Via Edoardo Orabona, 4, Bari, 70125 Italy; 2 D.E.T.O. University of Bari Medical School, Piazza Giulio Cesare, 11, Bari, 70124 Italy

**Keywords:** Deep learning, Convolutional neural network, Semantic segmentation, R-CNN, ADPKD, Magnetic resonance

## Abstract

**Background:**

The automatic segmentation of kidneys in medical images is not a trivial task when the subjects undergoing the medical examination are affected by Autosomal Dominant Polycystic Kidney Disease (ADPKD). Several works dealing with the segmentation of Computed Tomography images from pathological subjects were proposed, showing high invasiveness of the examination or requiring interaction by the user for performing the segmentation of the images. In this work, we propose a fully-automated approach for the segmentation of Magnetic Resonance images, both reducing the invasiveness of the acquisition device and not requiring any interaction by the users for the segmentation of the images.

**Methods:**

Two different approaches are proposed based on Deep Learning architectures using Convolutional Neural Networks (CNN) for the semantic segmentation of images, without needing to extract any hand-crafted features. In details, the first approach performs the automatic segmentation of images without any procedure for pre-processing the input. Conversely, the second approach performs a two-steps classification strategy: a first CNN automatically detects Regions Of Interest (ROIs); a subsequent classifier performs the semantic segmentation on the ROIs previously extracted.

**Results:**

Results show that even though the detection of ROIs shows an overall high number of false positives, the subsequent semantic segmentation on the extracted ROIs allows achieving high performance in terms of mean Accuracy. However, the segmentation of the entire images input to the network remains the most accurate and reliable approach showing better performance than the previous approach.

**Conclusion:**

The obtained results show that both the investigated approaches are reliable for the semantic segmentation of polycystic kidneys since both the strategies reach an Accuracy higher than 85%. Also, both the investigated methodologies show performances comparable and consistent with other approaches found in literature working on images from different sources, reducing both the invasiveness of the analyses and the interaction needed by the users for performing the segmentation task.

## Background

Autosomal Dominant Polycystic Kidney Disease (ADPKD) is a hereditary disease characterised by the onset of renal cysts that lead to a progressive increase of the Total Kidney Volume (TKV) over time. Specifically, ADPKD is a genetic disorder in which the renal tubules become structurally abnormal, resulting in the development and growth of multiple cysts within the kidney parenchyma [1]. The mutation of two different genes characterises the disease. The ADPKD type I, which is caused by the PKD1 gene mutation, involves the 85 - 90% of the cases, usually affecting people older than 30 years. The mutation of the PKD2 gene, instead, leads to ADPKD type II (affecting the 10 - 15% of the cases), which mostly regards children developing cysts already when in the maternal uterus and die within a year. HConsidering the clinical characteristics of the patients with PKD1 or PKD2 mutations, they are the same, even though the latter mutation is associated with a milder clinical phenotype and a later onset of End-Stage Kidney Disease (ESKD). In all the cases, the size of cysts is extremely variable, ranging from some millimetres to 4 - 5 centimetres [2].

Currently, there is not a specific cure for ADPKD and the TKV estimation over time allows to monitor the disease progression. Tolvaptan has been reported to slow the rate of cysts enlargement and, consequently, the progressive kidney function decline towards ESKD [3, 4]. Since all the actual pharmacological treatments aimed at slowing the growth of the cysts, the design of a non-invasive and accurate assessment of the renal volume is of fundamental importance for the estimation and assessment of the ADPKD progression over time.

There are several methods in the literature performing the TKV estimation; traditional methodologies, requiring imaging acquisitions, such as Computed Tomography (CT) and Magnetic Resonance (MR), include stereology and manual segmentation [5, 6]. Also, several studies tried to correlate this metric with body surface and area measurements in order to have a non-invasive estimation of TKV [7, 8]. Stereology consists in the superimposition of a square grid, with specific cell positions and spacing, on each slice of the volumetric acquisition (CT or MR). The bidimensional area obtained counting all the cells containing parts of the kidneys, interpolated with the other slices, considering the thickness of the acquisitions, allows obtaining the final three-dimensional volume. Manual segmentation, instead, requires the manual contouring of the kidney regions contained in every slice. Several tools supporting this task have been developed, introducing digital free-hand contouring tools or interactive segmentation systems to assist the clinicians while delineating the region of interest.

Considering both the phenotyping of the disease and the introduced approaches, the segmentation of biomedical images of kidneys is a tricky and troublesome task, strictly dependent on the human operator performing the segmentation, also requiring expert training. In fact, co-morbidities and the presence of cysts in neighbouring organs or contact surfaces make challenging achieving an accurate and standardised assessment of the TKV.

To reduce the limitations of the previous methodologies, both in time and performance, due to the manual interaction, several approaches for the semi-automatic segmentation of kidneys have been investigated such as the mid-slice or the ellipsoid methods, allowing to estimate the TKV starting from a reduced number of selected slices [9–11]. Although the reported methodologies are faster and more compliant than the previous ones, these are far from being accurate enough to be used in clinical protocols [12, 13].

In recent years, innovative approaches based on Deep Learning (DL) strategies have been introduced for the classification and segmentation of images. In details, deep architectures, such as Deep Neural Networks (DNNs) or Convolutional Neural Networks (CNNs), allowed to perform image classification tasks, detection of Regions Of Interest (ROIs) or semantic segmentation [14–17], reaching higher performance than traditional approaches [18]. The architecture of DL classifiers let avoiding the design of procedures for extracting hand-crafted features, as the classifier itself generally computes the most characteristic features automatically for each specific dataset. These peculiarities let DL approaches to be investigated in different fields, including medical imaging, signal processing or gene expression analysis [19–23].

Lastly, recent studies about imaging acquisitions for assessing kidneys growth suggested that MR should be preferred to other imaging techniques [24]. However, different research works allowed estimating TKV starting from CT images thanks to the higher availability of the acquisition devices and the more accurate and reliable measurement of TKV and the volume of cysts. On the other side, CT protocols for ADPKD are always contrast-enhanced using a contrast medium harmful for the health of the patient under examination; also, CT exposes the patients to ionising radiations. On these premises, the automatic, or semi-automatic, segmentation of images from MR acquisitions for improving the TKV estimation capabilities should be further investigated for improving the state-of-the-art performances.

**Fig. 1:**
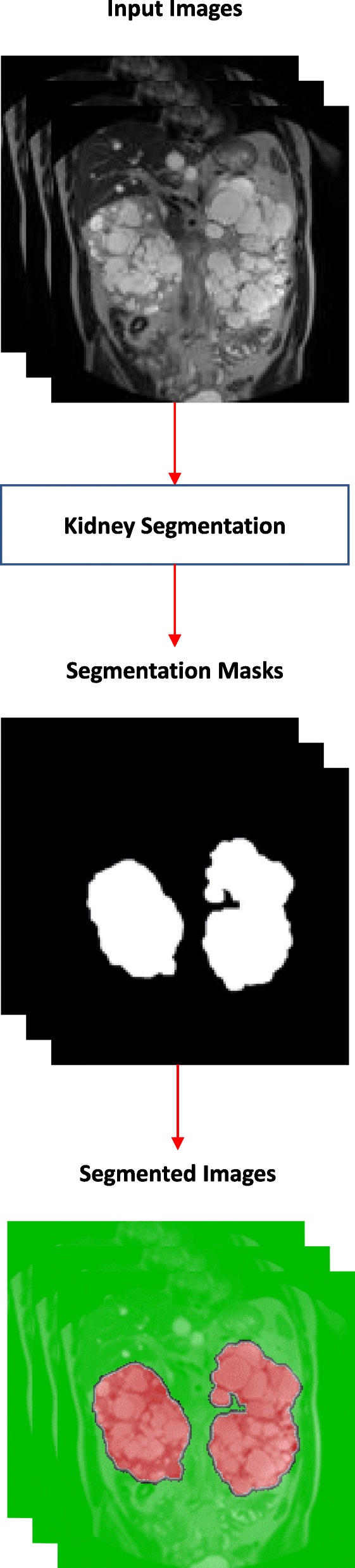
Workflow for the semantic segmentation starting from the full image

**Fig. 2:**
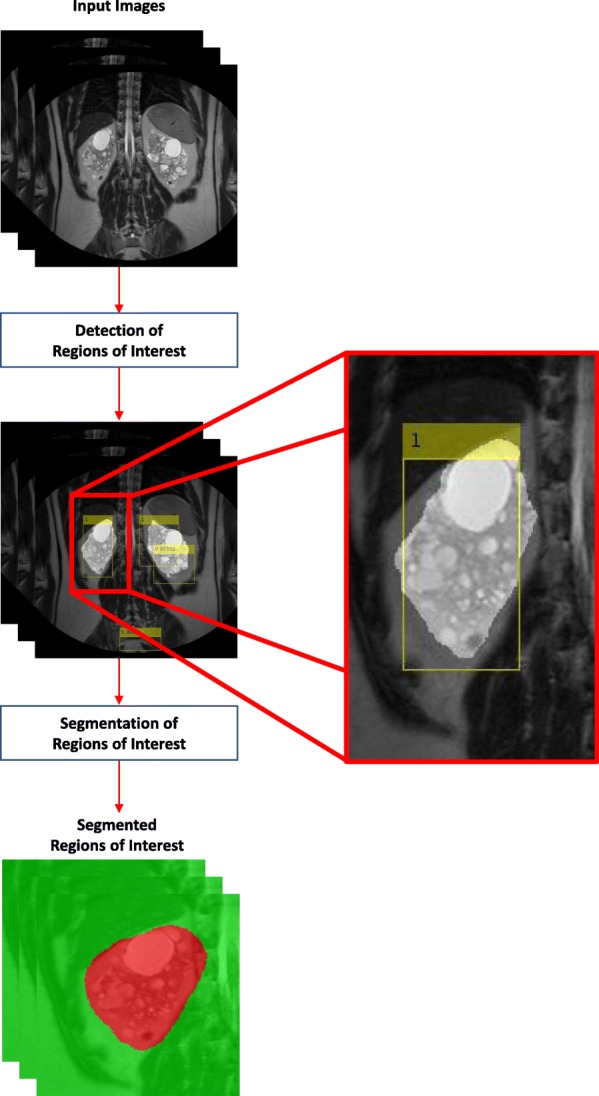
Workflow for the semantic segmentation of ROIs automatically detected with R-CNN

Starting from a preliminary work performed on a small set of patients [25], we present two different approaches based on DL architectures to perform the automatic segmentation of kidneys affected by ADPKD starting from MR acquisitions. Specifically, we designed and evaluated several Convolutional Neural Networks, for discriminating the class of each pixel of the images, in order to perform their segmentation; Fig. 1 represents the corresponding workflow. Subsequently, we investigated the object detection approach using the Regions with CNN (R-CNN) technique [26] to automatically detect ROIs containing parts of the kidneys, with the aim to subsequently perform the semantic segmentation only on the extracted regions; Fig. 2 shows a representation of the workflow implemented in this approach.

## Methods

### Patients and acquisition protocol

From February to July 2017, 18 patients affected by ADPKD (mean age 31.25 ± 15.52 years) underwent Magnetic Resonance examinations for assessing the TKV. The acquisition protocol was carried out by the physicians from the Department of Emergency and Organ Transplantations (DETO) of the Bari University Hospital.

Examinations for the acquisition of the images were performed on a 1.5 Tesla MR device (Achieva, Philips Medical Systems, Best, The Netherlands) by using a four-channel breast coil. The protocol did not use contrast material intravenous injection and consisted of:

- *Transverse and Coronal Short-TI Inversion Recovery (STIR) Turbo-Spin-Echo (TSE)* sequences (TR/TE/TI = 3.800/60/165 *ms*, field of view (FOV) = 250 *x* 450 *mm* (AP *x* RL), matrix 168 *x* 300, 50 slices with 3 *mm* slice thickness and without gaps, 3 averages, turbo factor 23, resulting in a voxel size of 1.5 *x* 1.5 *x* 3.0 *mm*^3^; sequence duration of 4.03 min);
- *Transverse and Coronal T2-weighted TSE* (TR/TE = 6.300/130 ms, FOV = 250 *x* 450 *mm* (AP *x* RL), matrix 336 *x* 600, 50 slices with 3 *mm* slice thickness and without gaps, 3 averages, turbo factor 59, SENSE factor 1.7, resulting in a voxel size of 0.75 *x* 0.75 *x* 3.0 *mm*^3^; sequence duration of 3.09 min);
- Three-Dimensional (3D) *T1-Weighted High Resolution Isotropic Volume Examination (THRIVE)* sequence (TR/TE = 4.4/2.0 ms, FOV = 250 *x* 450 *x* 150 *mm* (AP *x* RL *x* FH), matrix 168 *x* 300, 100 slices with 1.5 *mm* slice thickness, turbo factor 50, SENSE factor 1.6, data acquisition time of 1 min 30 s).

In this work, only the coronal T2-Weighted TSE sequence only was considered for the processing and classification strategies. In order to have the segmentation ground truth for all the acquired images, our framework included a preliminary step allowing the radiologists to manually contour all the ROIs using a digital tool specifically designed and implemented for this task.

**Fig. 3:**
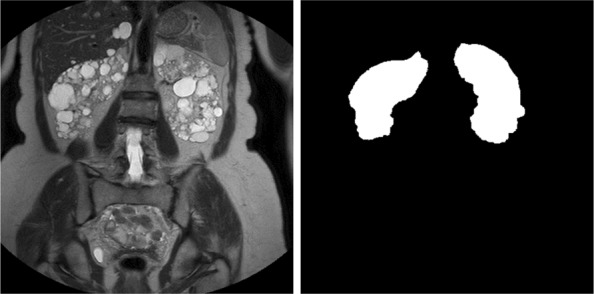
Example of an input image segmented manually; left: the representation of a DICOM image in greyscale; right: the mask obtained after the manual contouring of the selected slice

After the manual contouring of the kidneys, 526 images, with the corresponding labelled samples, constituted the working dataset; Fig. 3 represents an MR image with the corresponding labelled sample, where white pixels belong to the kidneys whereas the black ones include the remaining parts of the image.

### Segmentation approaches

Two different approaches based on DL techniques have been investigated to perform a fully-automated segmentation of polycystic kidneys without needing to design any procedure for the extraction of hand-crafted features. In details, the first approach allowed performing the semantic segmentation of the MR images, classifying each pixel belonging to the kidney or not; the second methodology, instead, allowed performing the detection of reduced areas containing the kidneys before their semantic segmentation.

#### Semantic segmentation

Semantic segmentation is a procedure allowing to perform the automatic classification of each pixel of images; thus, it is possible to classify each pixel of an image with a specific label. Although the segmentation of images is a well-established process in literature, counting a multitude of works and algorithms developed in several fields for different aims [27–29], the introduction and spread of DL architectures for performing this task, such as Convolutional Neural Networks, let image segmentation to regain interest in the scientific community [30, 31].

**Fig. 4:**
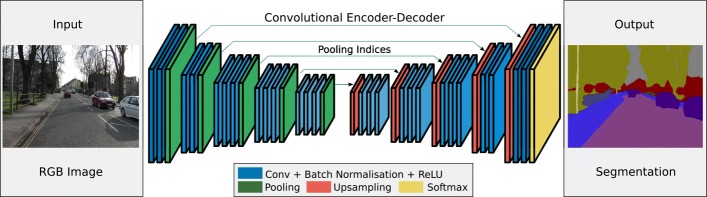
Encoder–Decoder architecture for SegNet [32]

According to different architectures designed in previous works, such as SegNet [32] and Fully Convolutional Network (FCN) [33], the CNNs performing semantic segmentation tasks show an encoder-decoder design, as the architecture represented in Fig. 4. Traditionally, this kind of classifier includes several encoders interspersed with pooling layers for downsampling the input; each encoder includes sequences of Convolutional layers, Normalisation layers and Linear layers. Based on the encoding part, there are specular decoders with up-sampling layers for reconstructing the input size. Finally, there are fully-connected neural units before the final classification layer able to label each pixel of the input image.

In this work, we designed and tested several CNNs architectures for the segmentation of the images. Since optimising the architecture of classifiers is still an open problem [34–36], often faced with evolutionary approaches, we decided to start from a well-known general CNN, the *VGG-16* [37], and modify its structure varying several parameters.

These included the number of encoders (and decoders), the number of layers for each encoder, the number of convolutional filters for each layer and the learner used during the training (i.e., SGDM - stochastic gradient descent with momentum, or ADAM [38]). All the investigated architectures included convolutional layers with kernels [3*x*3], stride [1 1] and padding [1 1 1 1] allowing to keep unchanged the dimensions of the input across each encoder; downsampling (and upsampling) was performed only in the max-pooling layers (upsampling layers for the decoder) having stride [2 2] and dimension [2*x*2].

The semantic segmentation of the input images took into account two classes: *Kidney* and *Background*. Considering the example reported in Fig. 3, the white pixels were labelled as *Kidney*, whereas the remaining pixels as *Background*.

For training the classifiers, also the dataset augmentation was performed according to recent works demonstrating the effectiveness of this procedure for improving the classification performance [31, 39, 40]; the following image transformations were randomly performed:

- horizontal shift in the range [-200; 200] pixels;
- horizontal flip;
- scaling with factor ranging in [0.5; 4].

Table 1 reports the configurations designed and tested for performing this task in terms of number of layers per encoder, number of convolutional filters per layer and applied learner. The table reports only the three configurations showing the higher performance among all the investigated architectures.

**Table 1:** Configurations designed and tested for the semantic segmentation of the full image

Network ID	Number of layers per encoder	Number of convolutional filters per layer	Learner
*VGG-16*	[2 2 3 3 3]	[64 128 256 512 512]	ADAM
*S-CNN-1*	[3 2 3 3 3]	[64 128 256 512 512]	ADAM
*S-CNN-2*	[3 2 3 3 3]	[96 128 256 512 512]	ADAM

Each layer is a sequence of a convolutional layer, a batch normalization layer and a ReLu layer

#### Regions with convolutional neural networks

Due to the presence of cysts in the organs near the kidneys and very similar structures located near the area of interest, which may affect the segmentation performance, we investigated a second approach based on the object detection strategy using R-CNN. In this approach, we designed a classifier for performing the automatic detection of smaller regions inside each input image to subsequently segment according to the procedure described in the previous section.

Object detection is a technique for finding instances of specific classes in images or videos. Like the semantic segmentation, also the object detection is a well-established process in literature employed in different fields [41, 42]. According to the literature, the CNNs for object detection include a region proposal algorithm, often based on EdgeBoxes or Selective Search [43, 44], as a pre-processing step before running the classification algorithm. Traditional R-CNN and Fast R-CNN are the most employed techniques [26, 45]. Recently, Faster R-CNN was also introduced, addressing the region proposal mechanism using the CNN itself, thus making the region proposal a part of the CNN training and prediction steps [46].

FAs for the previous approach, we investigated several CNN architectures for detecting areas containing the kidney, considering the Fast R-CNN approach. For creating the ground truth, the manual contour of each kidney was enclosed in a rectangular bounding box and used for training the network. Differently for the CNN aimed at performing the semantic segmentation, these architectures have only the encoding part, where each encoder includes Convolutional layers and ReLu layers. Each encoder ends with a max-pooling layer to perform image sub-sampling (size [3*x*3] and stride [2 2]). At the end of the encoding part, there are two fully-connected layers before the final classification layer. Table 2 reports the configurations designed and tested for the detection purpose (in this case too, the table reports only the three configurations that reached the higher performance).

**Table 2:** Configurations designed and tested for the CNN in the ROI detector

Network ID	Number of layers per encoder	Number of convolutional filters per layer	Learner
*R-CNN-1*	[3 3]	[32 32]	SGDM
*R-CNN-2*	[1 1]	[16 32]	SGDM
*R-CNN-3*	[3 3]	[64 32]	SGDM

Each layer is a sequence of a convolutional layer, and a ReLu layer

After designing the classifier for the automatic detection of the ROIs, the same architectures designed for the segmentation of the whole images (reported in Table 1), were considered for performing the semantic segmentation of the ROIs. Furthermore, since the detected ROIs might have different sizes, a rescaling procedure was performed to adapt all the ROIs to the size required by the CNN for the segmentation task. Images augmentation was performed, as well, considering the following image transformations:

- horizontal shift in the range [-25; 25] pixels;
- vertical shift in the range [-25; 25] pixels;
- horizontal flip;
- scaling with scale factor ranging in [0.5; 1.1].

## Results

This section reports the results for both the investigated approaches. In particular, we describe the performance obtained considering the R-CNN approach and subsequently, the results of the classifiers performing the semantic segmentation on both the full image and the ROIs automatically detected. The input dataset, which was constituted by MR images from 18 patients, was randomly split to create the training and test sets considering data from 15 and 3 patients, respectively. For improving the generalisation capabilities of the segmentation system, we performed a 5-fold cross-validation for the training the classifiers. The final segmentation on the images from the test set was obtained through the majority voting computed among the segmentation results from each trained classifier.

We considered several metrics for evaluating the classifiers; all the reported results refer to the performance obtained evaluating the networks only on the test set. *Accuracy* (Eq. 1), *Boundary F1 Score*, or BF Score, (Eq. 2) and *Jaccard Similarity Coefficient*, or Intersection over Union - IoU, (Eq. 3)were computed considering the number of instances of True Positives (*TP*), True Negatives (*TN*), False Positives (*FP*) and False Negatives (*FN*), where the Positive label corresponds to a pixel belonging to the *Kidney* class for the semantic segmentation approach, or to a ROI correctly detected (confidence > 0.8), for the R-CNN approach.

Regarding the semantic segmentation, the BF Score measures how close the predicted boundary of an object matches the corresponding ground truth; it is defined as the harmonic mean of the Precision (Eq. 5) and Recall (Eq. 6) values. The resulting score spreads in the range [0, 1], from a bad to a good match. The Jaccard Similarity Coefficient, instead, is the ratio between the number of pixels belonging to the Positive class classified correctly (*TP*) and the sum of the number of pixels belonging to the Positive class (*P* =*TP*+*FN*) and the Negative pixels wrongly predicted as Positive (*FP*). Regarding R-CNN performance, the *Average Precision* (Eq. 5) and the *Log-Average Miss Rate* were evaluated, considering the *Miss Rate* (MR) according to Eq. 4.

1 $$  Accuracy \;=\; \frac{TP+TN}{TP+TN+FP+FN}  $$

2 $$  Boundary\;F1\;Score \;=\; \frac{2*Precision*Recall}{Precision+Recall}  $$

3 $$  Jaccard\;Similarity\;Coefficient \;=\; \frac{TP}{TP+FP+FN}  $$

4 $$  Miss\;Rate \;=\; \frac{FN}{FN+TP}  $$

where:

5 $$  Precision \;=\; \frac{TP}{TP+FP}  $$

6 $$  Recall \;=\; \frac{TP}{TP+FN}  $$

### R-CNN performance

**Fig. 5:**
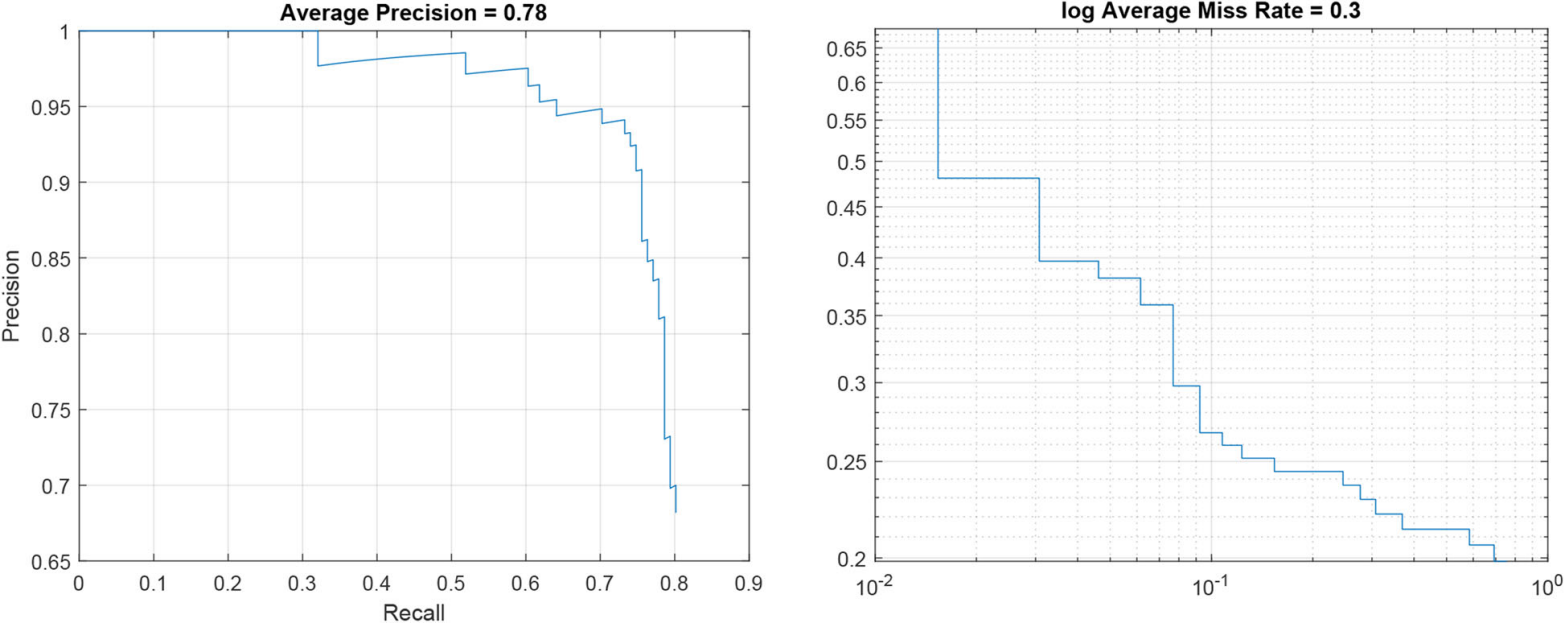
Precision – Recall plot and log Average Miss rate for R-CNN-1

**Fig. 6:**
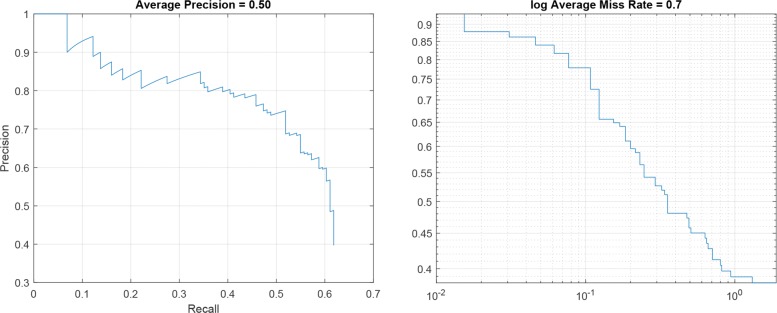
Precision – Recall plot and log Average Miss rate for R-CNN-2

**Fig. 7:**
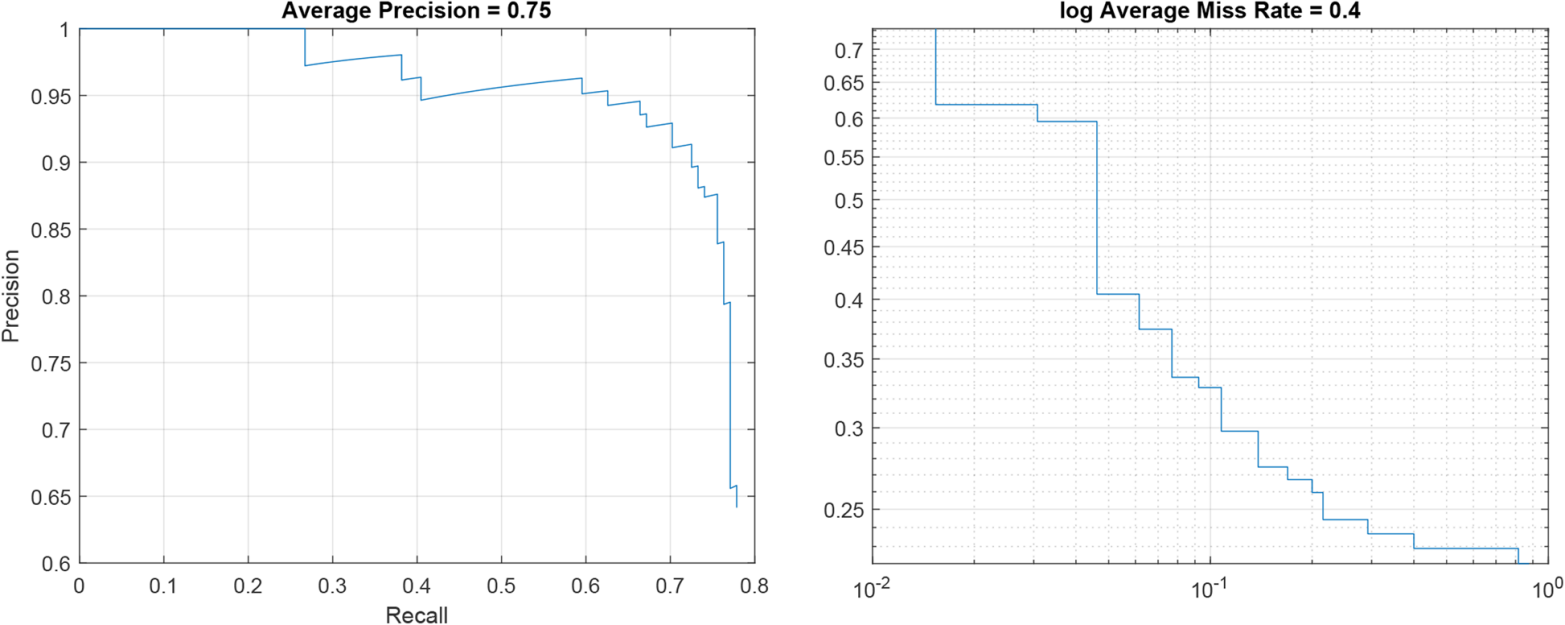
Precision – Recall plot and log Average Miss rate for R-CNN-3

**Fig. 8:**
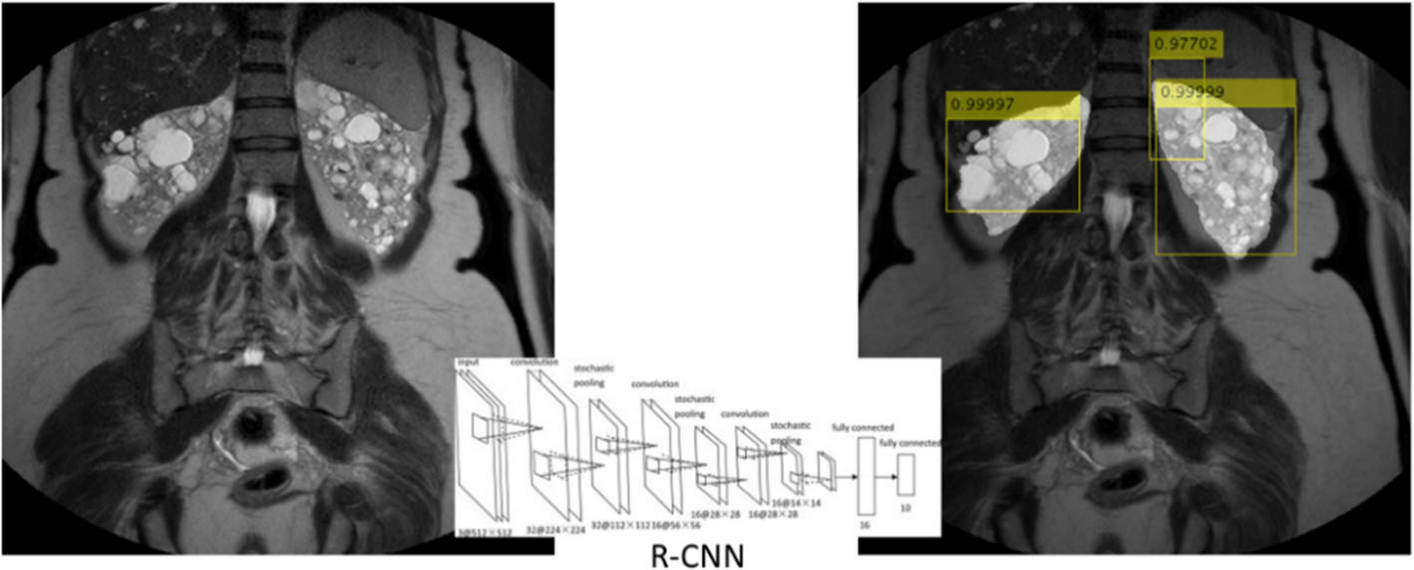
Results from R-CNN classifier. Input image is on the left; the image on the right contains squares on the detected ROIs, each one is associated with a score

For each R-CNN architecture reported in Table 2, the Precision-Recall plot, showing the Precision obtained at different Recall values, and the Log-Average Miss Rate plot, reporting how varies the miss rate at different levels of *FP* per image are represented. Specifically, Figs. 5, 6 and 7 show the plots for R-CNN-1, R-CNN-2 and R-CNN-3 respectively. Figure 8, instead, shows the result obtained performing the detection of kidneys on an image sample. As represented in the plots, the average Precision for R-CNN-1 and R-CNN-3 is higher than 0.75, also maintaining low the Log-Average Miss Rate.

Since the aim of detecting ROIs was the identification of regions with fewer Background pixels, respect to the whole image, for the subsequent semantic segmentation step, R-CNN-1 revealed to be the best candidate among all the analysed architectures. In fact, it reached a Recall value of about 0.8 with the Precision higher than 0.65, meaning that the classifier was able to detect the 80% of the ROIs containing the kidneys, but with a high number of False Positives. However, this was not a problem since the subsequent step of semantic segmentation would detect all the pixels belonging to the *Kidney* class.

### Semantic segmentation performance

Concerning the semantic segmentation, this section reports the performance obtained for the segmentation of both MR images and ROIs. Specifically, Table 3 shows the results obtained for each of the CNN architectures performing the semantic segmentation of the MR image, without performing any image processing procedure. As reported in the table, the architecture achieving the highest performance for the semantic segmentation of the full image is the *S-CNN-1*, showing an Accuracy higher than 88%.

**Table 3:** Performance indices for the classifiers working on MR images

Network ID	Mean accuracy	Weighted IoU	Mean BF score
*VGG-16*	0.88076	0.75288	0.41117
*S-CNN-1*	0.88359	0.76294	0.38205
*S-CNN-2*	0.79824	0.52781	0.38643

**Fig. 9:**
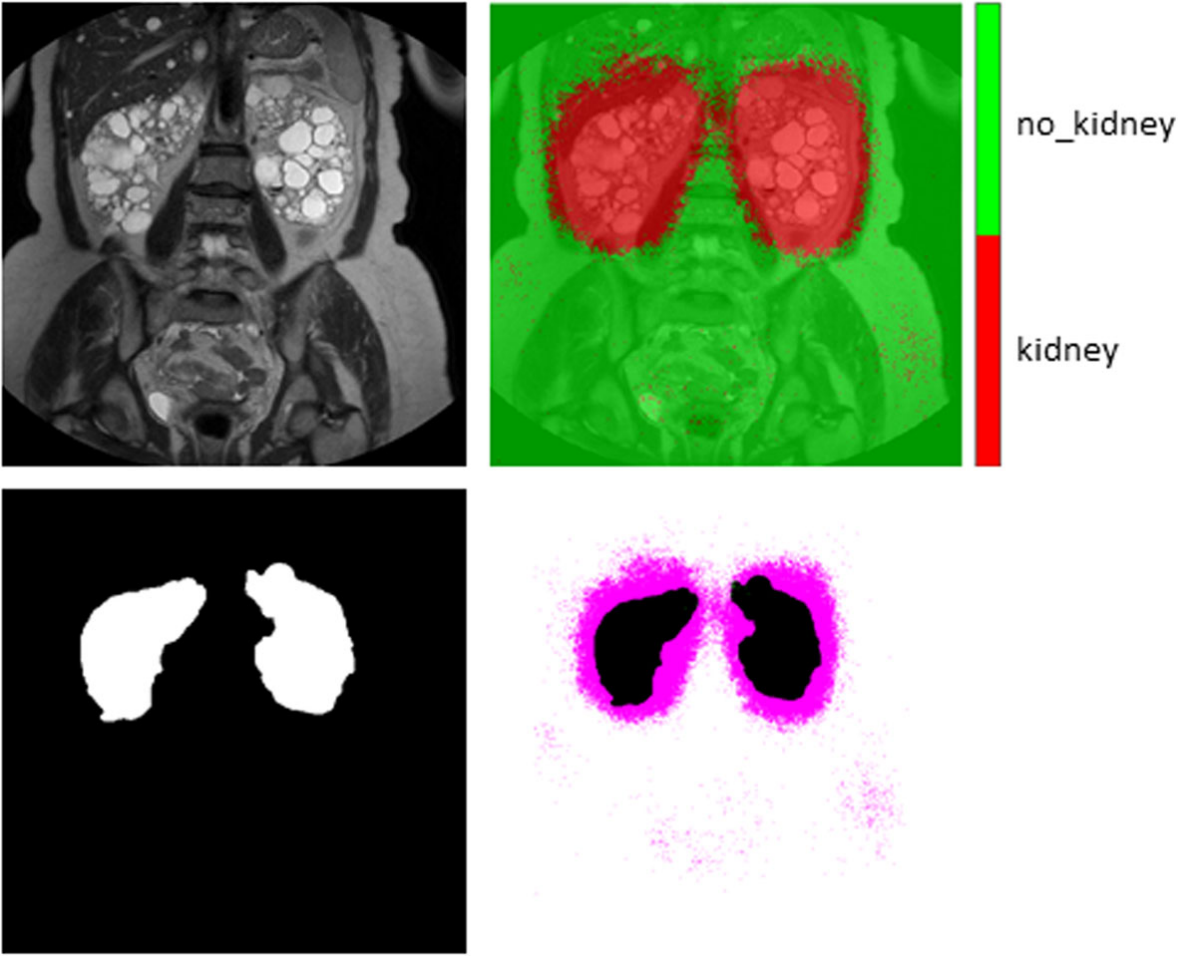
Result of the semantic segmentation considering an image sample. Top left: the MR slice represented in greyscale; top right: the segmentation result; bottom left: the ground-truth mask; bottom right: superimposition of the segmentation result to the ground-truth mask

The introduction of an additional layer into the first encoder of *VGG-16* architecture allowed the network to create a set of features more significant and discriminative than those generated by the others, leading to more accurate classification performance. Conversely, increasing the number of convolutional filters in the first layer of the first encoder of *S-CNN-1* did not improve the overall discrimination capabilities of the classifier. Table 4 reports the normalised confusion matrices obtained for the three considered cases in this approach, whereas Fig. 9 shows an example of the output generated by the implemented classifier performing the semantic segmentation of the MR images.

**Table 4:** Normalized Confusion Matrix for VGG-16, S-CNN-1 and S-CNN-2 segmenting the MR images for the test set

		VGG-16		S-CNN-1		S-CNN-2	
		True condition		True condition		True condition	
		*Positive*	*Negative*	*Positive*	*Negative*	*Positive*	*Negative*
Predicted	*Positive*	*0.96629*	*0.20477*	*0.96146*	*0.19428*	*0.96595*	*0.21611*
Condition	*Negative*	*0.03371*	*0.79523*	*0.03854*	*0.80572*	*0.03405*	*0.78389*

**Fig. 10:**
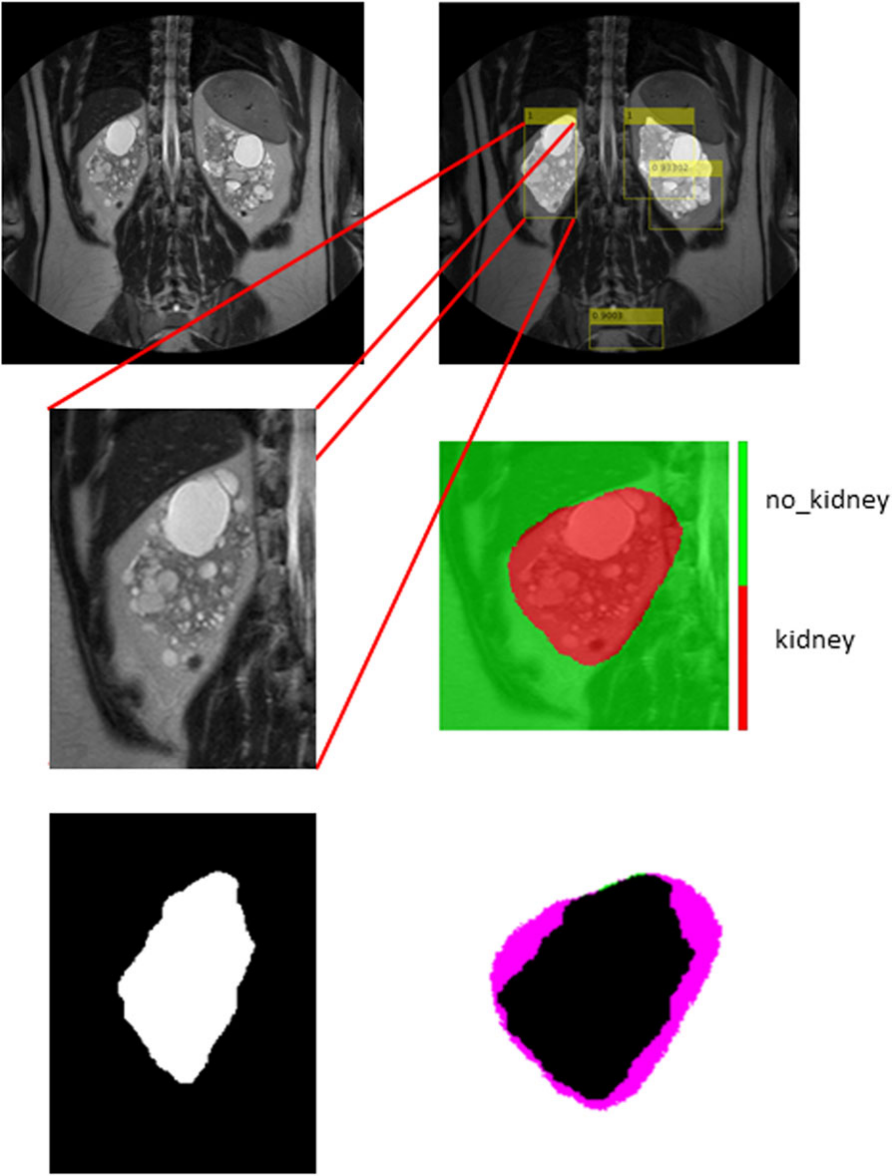
Example result for ROI detection and semantic segmentation. Top left: the MR slice represented in greyscale; top right: the R-CNN detection result; middle left: one of the detected ROIs; middle right the segmentation result; bottom left: the ground-truth mask for the considered ROI; bottom right: superimposition of the classification result to the ground-truth mask

As for the segmentation of the whole MR images, Table 5 reports the performance indices for the semantic segmentation of the ROIs automatically detected by the *R-CNN-1*, which showed the optimal trade-off in detecting ROIs considering the miss rate. As for the previous case, the *S-CNN-1* architecture allowed achieving the highest Accuracy in performing the semantic segmentation of the ROIs. Table 6 reports the normalised confusion matrices for all the classifiers investigated. Figure 10, instead, shows the results obtained for the semantic segmentation of ROI extracted from an image sample.

**Table 5:** Performance indices for the classifiers working on the ROIs

Network ID	Mean accuracy	Weighted IoU	Mean BF score
VGG-16	0.86016	0.75426	0.34828
S-CNN-1	0.8726	0.8540	0.4332
S-CNN-2	0.8550	0.82931	0.41515

**Table 6:** Normalized Confusion Matrix for VGG-16, S-CNN-1 and S-CNN-2 segmenting the ROIs detected by the R-CNN-1 from the MR images of the test set

		VGG-16		S-CNN-1		S-CNN-2	
		True condition		True condition		True condition	
		*Positive*	*Negative*	*Positive*	*Negative*	*Positive*	*Negative*
Predicted	*Positive*	*0.88781*	*0.16749*	*0.79742*	*0.05213*	*0.77762*	*0.06762*
Condition	*Negative*	*0.11219*	*0.83251*	*0.20258*	*0.94787*	*0.22238*	*0.93238*

## Discussion

In recent years, several works were proposed dealing with the segmentation of diagnostics images for assessing the ADPKD. Since the most used imaging procedure includes CT scans, most of the researches consider this kind of images in order to support the clinical assessment of the pathology. In some cases, the proposed approaches need minimum interaction by the user for the complete segmentation of the kidneys [47, 48]. Also, some procedures in literature dealt with the fully-automated segmentation of the images, some of them based on DL strategies [49, 50].

However, the proposed approaches for the automatic segmentation show several limitations, including the invasiveness from the contrast medium used for enhancing CT acquisitions [51], or rather the necessity of having an a-priori knowledge for the correct processing of the images [52]. In order to reduce the invasiveness of the imaging analysis, a preliminary investigation proposing a fully automated approach for the segmentation of non-contrast-enhanced CT images was proposed very recently, showing good performance on a reduced cohort of patients [53].

In this work, instead, the developed classification systems allowed to reach performances of about 80% of Accuracy in performing the segmentation of MR images, without using any procedure for contrast enhancement. However, the segmentation of the entire MR image revealed to be more reliable than those performed on the extracted ROIs. In fact, although the phase of extracting subregions from MR images showed an average Precision of 78%, it could still not find areas of interest, thus missing regions belonging to the kidneys.

According to the analysed literature, the reported results are consistent with other precursory investigations dealing with MR images, including the preliminary results presented in [25] on a reduced cohort of patients. Also, the proposed approaches overcome the limitations shown by manual or semi-automatic procedures in segmenting kidneys affected by ADPKD for evaluating diagnostics and prognostics parameters. In addition, the proposed methodologies did not use any contrast medium, thus without any harmful or potentially lethal ionising radiation for the patients.

## Conclusions

In this work, we investigated two strategies performing the automatic segmentation of MR images from people affected by ADPKD based on DL architectures. Both the designed strategies considered several Convolutional Neural Networks for classifying, between Kidney or Backgroud, all the pixels in the images.

In the first approach, we trained, validated and tested the classifiers considering the full MR image as input, without performing any procedure of image pre-processing. The second methodology, instead, investigated the object detection approach using the Regions with CNN (R-CNN) technique for firstly detecting ROIs containing parts of the kidneys. Subsequently, we employed (trained, validated and tested) the CNNs considered in the previous approach to perform the semantic segmentation on the ROIs automatically extracted by the R-CNN showing the most reliable performance.

The obtained results show that both the approaches are comparable and consistent with other methodologies reported in the literature, but dealing with images from different sources, such as CT scans. Also, the proposed approaches may be considered reliable methods to perform a fully-automated segmentation of kidneys affected by ADPKD.

In the future, the interaction among Deep Learning strategies and image processing techniques will be further investigated to improve the performance reached by the actual classifiers. Moreover, evolutionary approaches for optimising the topology of classifiers, or their hyper-parameters, will also be explored considering the acquired images in a three-dimensional way.

## Data Availability

The dataset employed for the current study is not publicly available due to restrictions associated with the anonymity of participants but could be made available from the corresponding author on reasonable request.

## Abbreviations

ADPKD: Autosomal dominant polycystic kidney disease
CNN: Convolutional neural network
CT: Computed tomography
DL: Deep learning
DNN: Deep neural network
ESKD: End-stage kidney disease
MR: Magnetic resonance
R-CNN: Regions with convolutional neural network
ROI: Region of interest
TKV: Total kidney volume
